# Integrating habitat‐masked range maps with quantifications of prevalence to estimate area of occupancy in IUCN assessments

**DOI:** 10.1111/cobi.14019

**Published:** 2022-11-16

**Authors:** Robert P. Anderson

**Affiliations:** ^1^ Department of Biology, City College of New York City University of New York New York New York USA; ^2^ Ph.D. Program in Biology Graduate Center, City University of New York New York New York USA; ^3^ Division of Vertebrate Zoology (Mammalogy) American Museum of Natural History New York New York USA

**Keywords:** AOO, detection, extinction, geographic distribution, IUCN Red List, occupied, risk, threatened, amenazada, AOO, detección, distribución geográfica, extinción, Lista Roja UICN, ocupada, riesgo

## Abstract

Estimates of species geographic ranges constitute critical input for biodiversity assessments, including those for the International Union for the Conservation of Nature (IUCN) Red List of Threatened Species. Area of occupancy (AOO) is one metric that IUCN uses to quantify a species’ range, but data limitations typically lead to either under‐ or overestimates (and unnecessarily wide bounds of uncertainty). Fortunately, existing methods in which range maps and land‐cover data are used to estimate the area currently holding habitat for a species can be extended to yield an unbiased range of plausible estimates for AOO. Doing so requires estimating the proportion of sites (currently containing habitat) that a species occupies within its range (i.e., prevalence). Multiplying a quantification of habitat area by prevalence yields an estimate of what the species inhabits (i.e., AOO). For species with intense sampling at many sites, presence–absence data sets or occupancy modeling allow calculation of prevalence. For other species, primary biodiversity data (records of a species’ presence at a point in space and time) from citizen‐science initiatives and research collections of natural history museums and herbaria could be used. In such cases, estimates of sample prevalence should be corrected by dividing by the species’ detectability. To estimate detectability from these data sources, extensions of inventory‐completeness analyses merit development. With investments to increase the quality and availability of online biodiversity data, consideration of prevalence should lead to tighter and more realistic bounds of AOO for many taxonomic groups and geographic regions. By leading to more realistic and representative characterizations of biodiversity, integrating maps of current habitat with estimates of prevalence should empower conservation practitioners and decision makers and thus guide actions and policy worldwide.

## Resumen

Estimaciones de las distribuciones geográficas de las especies constituyen insumos críticos para evaluaciones de la biodiversidad, incluyendo la Lista Roja de Especies Amenazadas de la Unión Internacional de la Conservación de la Naturaleza (UICN). El área de ocupación (AOO) es una métrica que usa la UICN para cuantificar la distribución de una especie aunque, típicamente, limitaciones en los datos disponibles hacen que métodos actuales produzcan subestimaciones o sobreestimaciones del área (y rangos de incertidumbre innecesariamente amplios). Afortunadamente, para producir un rango no sesgado de estimaciones plausibles para AOO se pueden desarrollar extensiones de métodos existentes en los cuales se usan mapas de distribución y datos de cobertura del suelo para estimar el área que efectivamente ofrece hábitat para una especie. Tal proceso requiere estimar la proporción de sitios que ocupa una especie dentro de su distribución (de las que actualmente proveen hábitat; i.e., prevalencia). Multiplicar una cuantificación del área con hábitat por la prevalencia resulta en un estimado del área que ocupa la especie (i.e., AOO). Para especies con muestreos intensivos en muchos sitios, la prevalencia se puede calcular utilizando conjuntos de datos de presencia/ausencia o el modelado de ocupación. Para otras especies se podrían usar datos primarios de biodiversidad (registros de la ocurrencia de una especie en un punto en el espacio y el tiempo) provenientes de iniciativas de ciencia ciudadana y colecciones de referencia de herbarios y museos de historia natural. En tales casos, estimaciones de la prevalencia de una muestra deben ser corregidas, dividiendo por la detectabilidad de la especie. Estimar la detectabilidad utilizando estas fuentes de datos amerita desarrollar extensiones de análisis de completitud de inventarios. Con esfuerzos para aumentar la calidad y disponibilidad de datos de biodiversidad en línea, el uso de prevalencia en el cálculo de AOO debe resultar en estimaciones más realistas y con rangos de incertidumbre reducidos para muchos grupos taxonómicos y regiones geográficas. Debido a que conducen hacía caracterizaciones más reales y representativas de la biodiversidad, técnicas que integran mapas de hábitat actual y estimaciones de prevalencia pueden empoderar a profesionales de la conservación y tomadores de decisiones, y así guiar acciones y políticas alrededor del mundo.

## INTRODUCTION

Quantifications of biodiversity and its change over time are key for answering outstanding questions in basic science and guiding policy regarding important applied issues (Scholes et al., 2008). The geographic distribution or range of a species constitutes a fundamental unit of biodiversity used in many assessments for management and conservation (Araújo et al., 2019; Hamilton et al., 2022; Jetz et al., 2019; Pereira et al., 2013). For most species, characterizations of the geographic range and changes to it constitute the principal information available to assess threat status for the International Union for the Conservation of Nature (IUCN) Red List of Threatened Species (Cazalis et al., 2022; IUCN, 2022; Palacio et al., 2021). Under these criteria, three categories of increasing risk indicate a species as threatened (vulnerable, endangered, or critically endangered). The IUCN applies two measures to quantify aspects of a species’ range: extent of occurrence (EOO) and area of occupancy (AOO). Both can be used to consider a species under criterion A (population size reduction) or criterion B (geographic range quantification). The EOO represents a measure of the geographic spread of the species’ range, usually calculated as the minimum convex polygon around sites of current known, inferred, or projected occurrence (defined in Table 1) (Brooks et al., 2019; IUCN, 2022). Complementarily, AOO quantifies the total areal extent of all 2×2 km cells that the species is known, inferred, or projected to occupy currently (IUCN, 2022) (Table 1).

**TABLE 1:** Glossary of key terms relevant in considering characterizations of the geographic range for assessment for the International Union for the Conservation of Nature (IUCN) Red List of Threatened Species following IUCN (2022) guidelines

Category	Term^a^	Definition
Risk level	threatened	species threatened by extinction as indicated by their listing as vulnerable, endangered, or critically endangered following IUCN (2022) guidelines
Kinds of sites	known sites	sites with confirmed extant (i.e., current) records of a species (IUCN, 2022)
	inferred sites	sites deduced as having a very high likelihood of presence for a species based on known sites and its habitat characteristics, dispersal capability, rates and effects of habitat destruction, and other relevant factors (IUCN, 2022)
	projected sites	sites spatially predicted to contain a species by habitat maps or models subject to additional considerations, including interpretation of potential habitat to indicate the areas as occupied (IUCN, 2022)
Geographic range quantifications	extent of occurrence (EOO)	measure of the spatial spread of the areas currently occupied by a species, usually calculated as the minimum convex polygon around sites of current known, inferred, or projected presence (excluding vagrant localities) (IUCN, 2022)
	area of occupancy (AOO)	quantification of the area of current habitat occupied by a species (total areal extent of all 2×2 km cells of known, inferred, or projected presence) (IUCN, 2022) (Figure 1)
	suitability (and suitable areas)	degree to which the environment is adequate for a species (Peterson et al., 2011); characterizations of suitability typically include abiotic variables and sometimes biotic factors and land‐cover information; for approaches that produce a continuous suitability prediction, a threshold is often applied to yield a binary characterization of suitable versus unsuitable
Frequency in nature and in biological samples	prevalence (true prevalence)	proportion of sites a species occupies across its range (Hanberry & He, 2013) (i.e., the probability of presence in occupancy modeling [MacKenzie et al., 2002]; when restricted specifically to suitable areas, prevalence equals the conditional probability of presence, given suitable conditions); multiplying the area suitable for a species by its prevalence yields area occupied
	sample prevalence (raw prevalence)	proportion of sites where a species has been detected across its range (compare with true prevalence, above); due to imperfect detection, a species’ sample prevalence (sometimes termed the *encounter rate* [Johnston et al., 2021]) is lower than its true prevalence
	detectability	chance of observing a species when it is present (i.e., conditional probability of detection, given presence) (Gu & Swihart, 2004; MacKenzie et al., 2002); dividing sample prevalence by detectability yields true prevalence
	inventory completeness	proportion of species detected to date out of the number estimated to be present (Colwell & Coddington, 1994; Moreno et al., 2018)

^a^ Order of related terms follows appearance in the text.

**FIGURE 1:**
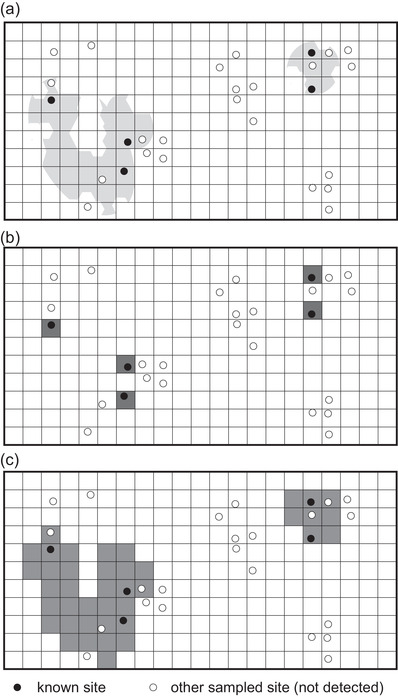
Schematic hypothetical illustration of existing methods to estimate area of occupancy (AOO) for assessment for the International Union for the Conservation of Nature (IUCN) Red List of Threatened Species following the approach explained in the IUCN (2022) guidelines (black dots, sites of known presence for a species; white dots, sites where sampling was conducted but did not detect the species due to its absence or inadequate sampling; grid, 2×2 km cells of standardized resolution for calculating AOO): (a) known and other sampled sites on a habitat‐masked range map (light gray) that indicates areas currently suitable for the species, (b) occupied‐cell method in which the species’ occupancy is assigned only to sites of known presence with current habitat (often leads to underestimates of AOO), (c) methods based on expert‐drawn or model‐based habitat‐masked range map that shows all areas currently suitable for the species across its distribution (Table 2) (often lead to overestimates of AOO, e.g., here at half the sampled sites in currently suitable areas, the species was not detected).

Ideally, both are considered in a species assessment, but EOO is used more often because of challenges inherent in calculating AOO given the limited occurrence data available for most species (the Wallacean shortfall) (Lomolino, 2004). Because of these limitations, existing methods for calculating AOO typically yield biased, implausible under‐ or overestimates (leading to unnecessarily wide bounds of uncertainty) (Figure 1 & Table 2). Nevertheless, AOO holds the key advantage of reflecting changes in species distributions more directly than EOO, for example, via remotely sensed information that characterizes land‐use change (Pettorelli et al., 2016). A summary of drawbacks for existing methods to calculate AOO and an outline of viable paths forward appear below—including necessary methodological development to allow widespread implementation. Building on methods that produce maps of places with habitat for a species, these solutions harness biodiversity data that either exist online now or whose quality and availability can be increased sufficiently for many taxa and regions via short‐ and medium‐term investments. Specifically, such biodiversity data can be used to estimate prevalence and detectability, allowing the conversion of a quantification of habitat area for a species into an unbiased, plausible estimate of what it occupies.

**TABLE 2:** Current and proposed methods for estimating the area of occupancy (AOO) for assessment for the International Union for the Conservation of Nature (IUCN) Red List of Threatened Species following the approach explained in the IUCN (2022) guidelines (Figure 1)^a^

Method	Description	Typical output	Assumption	Alternative when assumption unreasonable
Occupied cells (IUCN, 2022)	includes only sites a species is known to occupy currently	implausibly low lower bound	data derive from sufficient sampling for the species in all cells across its range	methods based on habitat‐masked range map
Habitat‐masked expert‐drawn range map (e.g., area of habitat) (Santini et al., 2019; Brooks et al., 2019)	masks expert‐drawn range map to remove areas that currently do not match the species’ elevational and land‐cover associations	implausibly high upper bound	species occurs in all suitable areas across its range	prevalence‐based conversion
Habitat‐masked, model‐based range map (Anderson & Martínez‐Meyer, 2004; Kass et al., 2021a)	masks model‐based range map (e.g., from species distribution model or ecological niche model based on climatic variables) to remove areas that currently do not match the species’ land‐cover or biotic associations	implausibly high upper bound	species occurs in all suitable areas across its range	prevalence‐based conversion
Prevalence‐based conversion of habitat‐masked range map (IUCN, 2022; methodologies proposed in this article)	adjusts quantification of suitable areas based on the proportion of habitat occupied; specifically, multiplies quantification based on habitat‐masked range map by the species’ prevalence (proportion of suitable sites that it occupies)	valid estimate (with plausible bounds determined by uncertainty of data and methods)	species’ prevalence homogeneous over its range (e.g., across habitats and suitability levels)	calculate prevalence stratified by such factors

^a^ Given that assumptions often are not met, the three current methods typically lead to either underestimates (occupied cells) or overestimates (two variations of habitat‐masked range maps). In contrast, the prevalence‐based conversion method builds on habitat‐masked range maps by integrating additional data to follow IUCN recommendations and produce an unbiased estimate of AOO (Figure 2). Instead of a habitat‐masked model‐based range map, statistical models can be made that include current land‐cover information as predictor variables (temporally matched with recent occurrence records).

## DRAWBACKS OF EXISTING METHODS FOR CALCULATING AOO

When determining the threat level for a species, IUCN guidelines call for a precautionary but realistic attitude to uncertainty, considering plausible lower and upper bounds for a metric rather than only the best estimate (IUCN, 2022). Lower and upper bounds around a best estimate should reflect uncertainty related to the data and methods of estimation employed. Nevertheless, the literature providing methods for estimating AOO has instead presented lower‐ and upper‐bound estimates based on the kinds of sites considered (Table 2). Such methods range from those quantifying a lower bound based only on known sites through others estimating an upper bound that includes all projected ones. However, these latter methods consider that all areas currently containing habitat within the species’ range indicate projected sites, without determining what subset is very likely to be occupied (Brooks et al., 2019; Kass et al., 2021a; Santini et al., 2019).

For the vast majority of species, these quantifications represent biased, implausible estimates that together bracket a range of uncertainty far wider than reasonable or necessary (Figure 1 & Table 2). At the lower extreme, the occupied‐cells method includes only sites that a species is known to occupy currently (assuming sufficient sampling for the species across its range) (Figure 1b & Table 2). This method yields accurate values when sampling is spatially dense (especially for range‐restricted species). However, for most species, it produces biased, implausible underestimates of the lower bound of AOO because most 2×2 km cells of the Earth have not been sampled adequately or at all for most taxonomic groups (i.e., the low spatial density of sampling). At the upper extreme, to circumvent the low spatial density of sampling, two methods estimate the areas currently suitable for a species by habitat masking of expert‐drawn or model‐based range maps, respectively (assuming the species’ presence in all areas holding habitat across its range) (Figure 1c & Table 2). However, these latter methods typically yield biased, implausible overestimates of the upper bound of AOO because they indicate all areas within the species’ range that are suitable but not necessarily occupied (Santini et al., 2019). Biased estimates and unnecessarily inflated bounds of uncertainty impede realistic threat assessment and balanced consideration of cost versus benefit for actions or policies.

The two habitat‐masking methods do not yield plausible estimates of AOO by themselves but nevertheless hold high utility for threat assessment. The most common method generally begins with an expert‐drawn range map and masks it to remove areas that currently do not match the species’ elevational and land‐cover (e.g., vegetational) associations (e.g., area of habitat procedure) (Table 2) (Brooks et al., 2019; Santini et al., 2019). Analogous masking options exist for aquatic species. The other method employs statistical models (often called species distribution models or ecological niche models) that use occurrence records and environmental variables (typically regarding climate) to identify areas potentially suitable for the species within a region lacking major dispersal barriers (Table 2) (Anderson & Martínez‐Meyer, 2004; Peterson et al., 2018). Such model‐based range maps can be processed to mask areas that either currently lack necessary land cover (Gavrutenko et al., 2021; Merow et al., 2022; Pettorelli et al., 2016) or where the distributions of key biotic interactors preclude the species’ presence (Kass et al., 2021a). Alternative to such postprocessing, when sufficient recent occurrence records exist, statistical models can include as predictor variables temporally matched information regarding land cover (Hamilton et al., 2022).

Nevertheless, even with appropriate consideration of variables reflecting land cover, range maps typically lead to biased, implausible overestimates of the sites a species is projected to occupy. Species are absent from many currently suitable areas within their ranges for a variety of reasons. These include hunting and overharvesting, unconsidered biotic interactors, inadequate patch size, high isolation of patches, and environmental and population‐demographic stochasticity. Accordingly, IUCN guidelines call for considering the proportion of habitat that the species is projected as very likely to occupy (IUCN, 2022). However, the literature has lacked sufficient information regarding how to accomplish this critical step for habitat‐masked range maps (Kass et al., 2021a).

## INTEGRATING DATA ON PREVALENCE AND DETECTABILITY

### Conceptual framework

**FIGURE 2:**
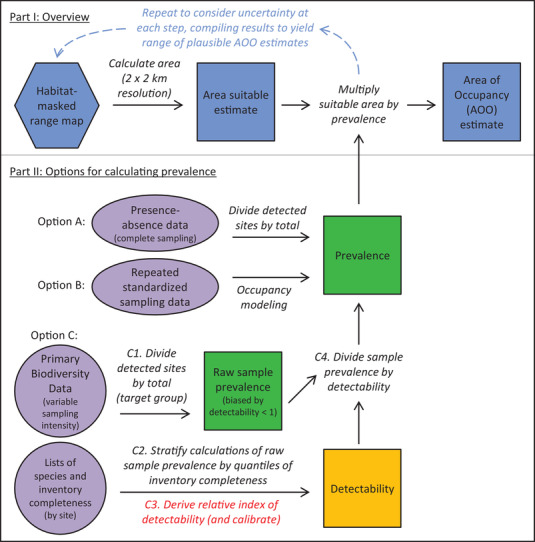
Illustration of the prevalence‐based conversion method to estimate the area of occupancy (AOO) for assessment for the International Union for the Conservation of Nature (IUCN) Red List of Threatened Species following the approach explained in the IUCN (2022) guidelines (terms defined in Tables 1 & 2): part I, an overview of the process (dashed arrows, process repeated as necessary); part II, three options for calculating prevalence (feeds back to part I) (ovals and circles, necessary data inputs; hexagon, habitat‐masked range map [input to the process]; squares, quantitative estimates produced; option A, detectability equals 1; option B, detectability estimated in the process of calculating prevalence; option C, detectability estimated from primary biodiversity data and inventory completeness; part requires future development [red, C3]).

Unbiased values of AOO can be achieved by estimating a species’ prevalence and using that information to extend existing methods that quantify current habitat (Figure 2 & Table 2). The IUCN guidelines for AOO indicate that quantifications of suitable areas “need to be adjusted (using an estimate of the proportion of habitat occupied) to produce a valid estimate” (IUCN, 2022: 60). Multiplying the quantification of habitat area by an estimate of the species’ prevalence (how frequently it is present within the range) (Hanberry & He, 2013) (Table 1) does just that, indicating the area projected to be occupied (Kass et al., 2021a). Consideration of prevalence can reflect the overall effect of many latent factors that preclude a species’ presence in suitable areas within its range without modeling them explicitly. Integrating information regarding prevalence can yield an unbiased best estimate of AOO, as well as plausible lower and upper bounds around it (IUCN, 2022).

Estimating a species’ prevalence involves using information regarding detectability. The prevalence of a species in nature is the proportion of sites that it occupies across its range (Hanberry & He, 2013; Jiménez‐Valverde et al., 2009; but see Ficetola et al. [2015] for different usage). In particular, a species’ prevalence in suitable areas within its range (conditional probability of presence, given suitable conditions) constitutes the key conversion factor needed to yield an unbiased estimate of AOO (Figure 2 & Tables 1 & 2). Because prevalence typically varies across environments, ideally it should be estimated separately for different habitat types or suitability levels—averages over the parts of the map corresponding to each category. However, an unbiased calculation of prevalence requires information regarding detectability (Figure 2). Detectability is the chance of observing a species with particular sampling techniques when it indeed occupies a site (conditional probability of detection, given presence) (MacKenzie et al., 2002) (Table 1); detectability also varies across environments and among species. Without considering detectability, raw estimates of prevalence (sometimes termed the *encounter rate*) will be biased—lower than reality—for species that are hard to detect; such biases falsely indicate species as rarer than they really are (Johnston et al., 2021). For threatened‐species assessment, using prevalence to convert an estimate of the area suitable for a species into quantification of what it occupies constitutes a fusion of the occupied cells and habitat‐masked range‐map methods for calculating AOO (Table 2), along with additional biodiversity data regarding sampling effort. More generally, it represents a crossover between species distribution modeling and occupancy–detectability modeling, fields that have developed rather independently in recent years.

### How to calculate unbiased estimates of prevalence and detectability

Existing methods can be employed to quantify prevalence for some species, and analogous analyses could be possible for many taxonomic groups and geographic regions with further methodological development and expanded availability of high‐quality occurrence data. Three options exist, and the appropriate method depends on the data available. Typically, the original information used to generate a map of suitable areas will constitute a subset of that needed to estimate prevalence. Two clear possibilities exist and are possible today for groups of species enjoying high‐intensity sampling (even with low spatial density) (Figure 2). In the first case, exhaustive inventory efforts conducted at many suitable sites across a species’ range eliminate the need to consider detectability (option A in Figure 2). In such situations, the resulting presence–absence data set allows straightforward calculation of prevalence at suitable sites within the species’ range (number of places where the species has been detected, divided by total sites sampled), ideally stratified by habitat type or suitability level. Alternatively, in the case when exhaustive inventories do not exist but repeated standardized sampling has occurred at particular sites, occupancy modeling can be employed (option B in Figure 2) (MacKenzie et al., 2002; Royle et al., 2005; Strimias‐Mackey et al., 2020). Occupancy models calculate detectability for a species and take it into account in estimating the probability of presence (which equates to prevalence, assuming homogeneity among sites) (MacKenzie et al., 2002). Such models can characterize differences in detectability and prevalence across habitat types or suitability levels. Nevertheless, these two situations with high‐intensity sampling remain restricted to relatively few taxonomic groups and geographic regions (Strimias‐Mackey et al., 2020).

In the third case of groups of species where sampling not only has low spatial density but also varies in intensity across sites, future methodological development merging information from inventory‐completeness analyses could allow the use of data from citizen‐science initiatives and the research collections of natural history museums and herbaria (option C in Figure 2). Primary biodiversity data consist of individual records of species with locality, date, and taxonomic identification (Soberón & Peterson, 2004). The primary biodiversity data available online from such sources and aggregators, such as the Global Biodiversity Information Facility (Heberling et al., 2021), vary in quality; uncertainties of georeferences and taxonomic identifications seldom are quantified. Nevertheless, they hold great promise when documented, cleaned, and used wisely (Amano et al., 2016; Anderson, 2012; Anderson et al., 2020; Velásquez‐Tibatá et al., 2019). The heterogeneous sampling efforts that led to these data differ in intensity and techniques, and the spatial density of sampling varies across regions. However, with important caveats regarding detectability discussed below, occurrence data for all species that are observed with the same techniques (together termed the target group) can be used to quantify the sampling effort relevant for any particular one of them (the focal species) (Anderson, 2003; see also Lobo et al., 2018; Phillips et al., 2009). For example, in the Neotropics, typical sampling efforts for small nonvolant mammals might yield records of marsupials and rodents with body sizes below approximately 100 g—a target group useful for characterizing the spatial biases of relevant sampling efforts. Taking advantage of such records to estimate prevalence requires two assumptions: similar sampling techniques for the target group are employed at sites across suitable areas and uniform detection probability for a given species exists across suitable areas (Anderson, 2003). Although sampling techniques vary across individual sites, the use of target groups requires only that no systematic bias in them exists across space. Stratifying calculations of prevalence and detectability by habitat type and suitability levels should ameliorate departures from these assumptions.

With future methodological development of one key step described below, information derived from primary biodiversity data for an appropriate target group could be used to estimate a species’ prevalence and detectability (option C in Figure 2). For an idealized species with perfect detectability, prevalence at the subset of sites within its range that held suitable conditions at the time of sampling simply equals the number of places where it was detected divided by the total number of such sites with records of any species of the target group. However, detectability seldom reaches 1 for any species and sampling technique, often being much lower (Gu & Swihart, 2004; MacKenzie et al., 2002). Therefore, estimates of prevalence in a sample will be biased—lower than reality—if no correction is made for imperfect detection, and underestimates will be more extreme for species that are progressively harder to detect (step C1 in Figure 2). Sometimes termed the encounter rate, sample prevalence is equal to the species’ true prevalence multiplied by its detectability (Johnston et al., 2021). Therefore, a species’ true prevalence equals sample prevalence divided by detectability (analogous to corrections for abundance estimators using count data) (MacKenzie et al., 2002). Hence, when using primary biodiversity data from a target group, estimates of a species’ sample prevalence should be corrected (by dividing by its detectability) to gain an unbiased estimate of prevalence (with sample prevalence constrained to less than detectability) (step C4 in Figure 2).

To approximate this last piece of the puzzle—detectability—with primary biodiversity data, extensions of inventory‐completeness analyses at many individual sites provide a path forward meriting development (option C in Figure 2). Conducted separately at individual sites, such analyses indicate the proportion of species observed to date, out of the total estimated to be present. Hence, they quantify the level of inventory completeness of the community (Table 1) (Colwell & Coddington, 1994) (see also related term sample coverage [Chao & Jost, 2012]). Various ways exist to do so (Lobo et al., 2018; Moreno et al., 2018), often by comparing the particular species observed at a single site among given units of sampling effort (e.g., different days or traplines) (Anderson et al., 2012).

Detectability then could be approximated by comparing (across sites) lists of the particular species observed and the level of inventory completeness (option C in Figure 2). First, sample prevalence could be calculated for each species in parts of its range holding habitat at the time of sampling, stratifying calculations by quantiles of inventory completeness (step C2 in Figure 2). This would indicate which species are detected easily (even at sites with low inventory completeness) versus those with low detectability (observed only at sites nearing a coplete inventory). For example, when plotting multiple species on a graph of sample prevalence (y‐axis) versus inventory completeness (x‐axis), species with low detectability should show a steep positive slope, whereas those with high detectability would display little increase. Second, and constituting the one key step that requires development, at least a relative index of detectability across species then could be derived from such comparisons and scaled to range from 0 to 1 (step C3 in Figure 2) (e.g., based on comparisons among slopes; estimating intercepts; or borrowing from graphical methods, such as calibration plots) (Johnston et al., 2021; Phillips & Elith, 2010). When quantifications of absolute detection probability exist from other sources for some of the species (or could be borrowed from related taxa and similar habitat types), relative detectability values could be calibrated to yield absolute estimates for all of them (step C3 in Figure 2). Given enough sites, these analyses could be refined by considering habitat type and suitability level. In the first implementations of techniques to estimate prevalence with detectability values approximated from primary biodiversity data, results should be compared with estimates derived from other analyses (e.g., occupancy modeling for taxa and regions with appropriate data). This pathway (option C in Figure 2) would allow harnessing extensive primary biodiversity data, which for most species likely will lead to more realistic estimates than relying on a small subset of data amenable to occupancy modeling (option B in Figure 2) (Strimias‐Mackey et al., 2020).

### Best estimates, uncertainty, and scaling

Quantifying prevalence to extend approaches based on habitat‐masked range maps will allow lower and upper bounds of AOO to be based on uncertainty related to data and methods of estimation. Using a data‐driven approximation of a species’ prevalence to convert the quantification of the current habitat area into the subset projected to be occupied can yield an unbiased estimate of AOO (Figure 2). By extension, considering the uncertainty associated with the myriad issues involved in making such estimates could lead to plausible lower and upper bounds around a best estimate. These issues include uncertainty related to occurrence records, environmental variables, expert opinions, copious aspects of statistical modeling, and calculations of prevalence and detectability (Anderson, 2012; Araújo et al., 2019; Wieczorek et al., 2004; Zurell et al., 2020). Collating all plausible results and employing Bayesian statistics represent viable alternative paths to do so. Regarding uncertainty, IUCN guidelines prescribe a risk tolerance slightly lower than midway between precautionary and evidentiary attitudes (precautionary: classify a species as threatened unless it clearly is not; evidentiary: only classify a species as threatened if it clearly is). Consistent with this, the guidelines suggest employing a 90% confidence interval to delimit the plausible range of a metric (or 90% credible interval for Bayesian approaches) (IUCN, 2022).

Importantly, estimates of prevalence and detectability should be calculated at the same spatial resolution as the habitat‐masked range map (2×2 km for AOO). Prevalence (including information regarding detectability) generally is estimated at the scale of a site. Hence, before being used to convert the quantification of habitat area into AOO, estimates of prevalence must be transformed from the proportion of sites occupied into the proportion of 2×2 km cells occupied. With extremely dense sampling (many sites in each 2×2 km grid cell), even low values of site‐level prevalence will correspond to 100% grid‐cell prevalence, a strong mismatch between scales. At the other extreme, with sparse sampling (the vast majority of 2×2 km cells lacking any sampling), site‐level prevalence will approximate that of grid cells. Fortunately, the species most needing consideration of prevalence (typically with no sites sampled in most grid cells) correspond to the latter situation in which estimates of prevalence at the site‐ and grid‐cell scales converge.

## SIMPLE HYPOTHETICAL ILLUSTRATION

**FIGURE 3:**
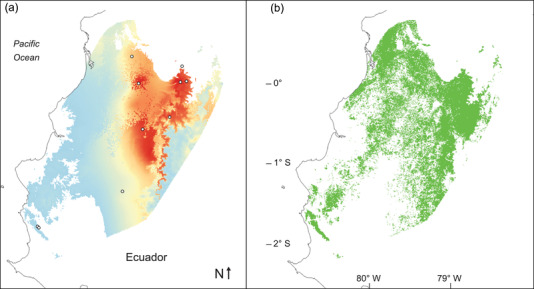
Hypothetical example for the forest‐restricted Ecuadorean spiny pocket mouse (*Heteromys teleus*) showing how the proposed prevalence‐based conversion method for calculating the area of occupancy (AOO) could improve assessment for the International Union for the Conservation of Nature (IUCN) Red List of Threatened Species following IUCN (2022) guidelines: (a) continuous suitability range, showing stark gradients within the species’ range (warmer colors indicate higher suitability) and (b) binary habitat‐masked range map (green) after considering current forest cover (Kass et al., 2021a). Both maps are at the native resolution of environmental data (not 2×2 km as used for calculating AOO). Existing methods (Table 2) yield a wide range of implausible estimates for AOO, designating the species as either endangered or only near threatened. In contrast, the prevalence‐based conversion method could yield an unbiased estimate of the area occupied. For example, a hypothetical uniform value of 0.15 (90% CI 0.10−0.20) for prevalence across all currently suitable areas in the range of *H. teleus* would indicate the species is threatened (vulnerable). Quantifying prevalence stratified by suitability level would aosimprove estimates of AOO (most deforestation in the range of *H. teleus* corresponds to areas of low modeled suitability).

Hypothetical examples for a real species of conservation concern illustrate how consideration of prevalence could improve threat assessments. The Ecuadorian spiny pocket mouse (*Heteromys teleus*) is a forest‐restricted species occurring in a region with high deforestation and sparse biological sampling (Anderson & Jarrín‐V., 2002) (Figure 3). Recent estimates of AOO range over three degrees of magnitude, from a lower bound of 36 km^2^ based on occupied cells (nine known sites currently holding forests) to 18,544 km^2^ as an upper bound provided by a habitat‐masked range map from a statistical model (with uncharacterized uncertainty) (Kass et al., 2021a) (Figure 3). That lower‐bound estimate would designate the species as threatened (endangered B2, far below the threshold of <500 km^2^; two necessary subrequirements of criterion B likely met). However, it surely underestimates the lower bounds of AOO, given the extremely low spatial density of sampling in the region. In contrast, the upper‐bound estimate would correspond only to near‐threatened (B2). Although prevalence and detectability for *H. teleus* remain unknown, the species has not been observed frequently via sampling in suitable areas (Anderson & Jarrín‐V., 2002; Jarrín E., 2013; Kass et al., 2021a). Hence, the estimate of AOO based on the habitat‐masked range map presumably overestimates its plausible upper bound.

The prevalence‐based conversion method could improve the assessment of *H. teleus* under criteria A or B (IUCN, 2022) (Figure 3). As a simple hypothetical example under criterion B (geographic range quantification), imagine an estimated value of 0.15 (and 90% CI of 0.1−0.2) for the species’ prevalence across all suitable areas with remaining forest. Assuming that the model‐based quantification of suitable, forested areas had no error, multiplication by the calculation of prevalence would yield an AOO best estimate of 2782 km^2^ and a plausible range of 1854−3709 km^2^. That would indicate that the species is threatened (vulnerable B2, < 2000 km^2^). However, a moderately higher hypothetical value of 0.30 (and 90% CI of 0.25−0.35) would generate an AOO best estimate of 5563 km^2^ and a plausible range of 4636−6490 km^2^, far above the threshold for any threatened category.

Alternative to these examples, hypothetical consideration of prevalence under criterion A (population size reduction) illustrates the value of stratifying calculations by suitability level. A comparison of suitable areas before and after masking by forest cover indicates a 53% decline, which would (barely) qualify the species as endangered (A2[c], ≥ 50%). However, most deforestation within the species’ range has affected extensive areas of low modeled suitability (Kass et al., 2021a) (Figure 3), where the species likely has a lower prevalence. Therefore, consideration of prevalence by suitability level presumably would lead to an estimated decline in AOO less extreme than the 50% threshold for endangered—perhaps not even meeting that for vulnerable (≥ 30%, A2[c]). Under either criterion, real‐world implementations of statistical models and prevalence‐based conversion should also characterize all relevant sources of uncertainty to establish lower and upper bounds around best estimates. More generally, they should follow IUCN guidelines for statistical models (IUCN, 2022) and consider proposed standards for species distribution models used in biodiversity assessments (Araújo et al., 2019; Sofaer et al., 2019).

## IMPLEMENTATION AND OUTLOOK

In comparison with current methods for calculating AOO, two general tendencies should emerge with the prevalence‐based conversion of areal quantifications derived from habitat‐masked range maps (Figures 1, 2, 3 & Table 2). First, this method will tend to lead to larger areas (and lower threat levels) than that based on occupied cells. The degree of increase in areal estimates will be greatest for species with the sparsest sampling (lowest spatial density). Second, the prevalence‐based conversion will tend to lead to smaller areas (and higher threat levels) than the methods that quantify suitability from habitat‐masked range maps. The degree of decrease in areal estimates will be greatest for species with the lowest prevalence. Overall, in implementing prevalence‐based conversion, reductions in the spread between lower and upper bounds should be most substantial for sparsely sampled species with low prevalence.

Although most of the underlying methodologies highlighted here already exist, widespread implementation of the prevalence‐based conversion method will involve surmounting challenges related to methodological development as well as data quality and availability. For some taxonomic groups and geographic regions, appropriate data from high‐intensity sampling allow immediate use. For others, where sampling intensity varies greatly, future development is needed to calculate detectability based on primary biodiversity data and inventory‐completeness analyses. In addition, the conservation need for IUCN Red List assessments provides tangible justification for investments to improve the quality and availability of online primary biodiversity data. Relevant efforts include ongoing digitization, further georeferencing, and improved taxonomic identifications—with quantifications of the uncertainty associated with the latter two (Anderson et al., 2020; Beaman & Cellinese, 2012; Hedrick et al., 2020). The level of georeferencing uncertainty permissible for a given analysis will vary according to the natural history of the species and the spatial resolution of the relevant environmental data (e.g., land‐cover information). In contrast, presumably only records with low uncertainty of taxonomic identification should be used. Notably, the primary biodiversity data needed for quantifying prevalence and detectability constitute the same information used to build the statistical models that represent one source of habitat‐masked range maps, although inventory‐completeness analyses likely will require greater temporal precision (exact date of the record). The eventual production of well‐documented code and user‐friendly interfaces to conduct all of these analyses should facilitate their uptake (Cazalis et al., 2022; Kass et al., 2021b, 2018).

Such analytical and data‐related advancements for biodiversity conservation should also promote a positive feedback loop with basic science. Improved range estimates would facilitate many uses in macroecology and biogeography and applications to other problems of importance to society, for example, invasive species and zoonotic diseases (Graham et al., 2004; Johnson et al., 2019; Thompson et al., 2021). In particular, mapped values of prevalence would represent an added dimension to range maps, which typically provide only relative suitability information (Guillera‐Arroita et al., 2014). Additionally, the estimated prevalence for individual species would enable probabilistic quantifications of richness, endemism, and related measures, which today usually derive from binary maps (Guisan & Rahbek, 2011; Paz et al., 2021). Such characterizations of assemblage‐level metrics across space would also contribute to applied uses (Pettorelli et al., 2016; Proença et al., 2017). If successful, this article will catalyze advances that promote more realistic and representative biodiversity information useful both for basic science and to guide actions and policy in a rapidly changing world.
